# Pleural sarcoidosis diagnosed on the basis of an increased CD4/CD8 lymphocyte ratio in pleural effusion fluid: a case report

**DOI:** 10.1186/s13256-015-0656-y

**Published:** 2015-08-14

**Authors:** Toru Kumagai, Yasuhiko Tomita, Takako Inoue, Junji Uchida, Kazumi Nishino, Fumio Imamura

**Affiliations:** 1 Department of Thoracic Oncology, Osaka Medical Center for Cancer and Cardiovascular Diseases, 1-3-3 Nakamichi Higashinari-ku, Osaka, 537-8511 Japan; 2 Department of Pathology, Osaka Medical Center for Cancer and Cardiovascular Diseases, 1-3-3 Nakamichi Higashinari-ku, Osaka, 537-8511 Japan

**Keywords:** CD4/CD8 lymphocyte ratio, Pleural effusion, Pleural sarcoidosis, Sarcoidosis, Thoracentesis

## Abstract

**Introduction:**

Pleural effusion induced by sarcoidosis is rare, and pleural sarcoidosis is often diagnosed by thoracoscopic surgery. The diagnosis of pleural sarcoidosis using thoracentesis may be less invasive when sarcoidosis is already diagnosed histologically in more than one organ specimen. Here we report the case of a 64-year-old woman with pleural sarcoidosis diagnosed on the basis of an increased CD4/CD8 lymphocyte ratio in pleural effusion fluid obtained by thoracentesis. This case report is important because it highlights the usefulness of the CD4/CD8 lymphocyte ratio in pleural effusion as an indicator of pleural involvement of sarcoidosis.

**Case presentation:**

A 64-year-old Japanese woman visited our hospital with an initial symptom of dyspnea on exertion for a period of 4 months. Chest computed tomography showed bilateral hilar and multiple mediastinal lymphadenopathy, multiple small nodular shadows in her bilateral lungs, small nodular shadows along the interlobar pleura, and bilateral pleural effusion. Her serum angiotensin-converting enzyme and soluble interleukin-2 receptor levels were elevated. Histological analysis of a resected subcutaneous nodule, and biopsy specimens from a right mediastinal lymph node and from her right lung revealed non-caseous epithelioid granulomas. Her bronchoalveolar lavage fluid exhibited a predominance of lymphocytes together with an increase in the CD4/CD8 lymphocyte ratio. The lymphocytic predominance and the increased CD4/CD8 lymphocyte ratio were also detected in the right-sided pleural effusion fluid obtained by thoracentesis. We diagnosed sarcoidosis with pleural involvement. Because pleural effusion did not resolve spontaneously and her symptom of dyspnea on exertion worsened, corticosteroid therapy was initiated, which ameliorated the sarcoidosis and the pleuritis.

**Conclusions:**

Analysis of the CD4/CD8 lymphocyte ratio in pleural effusion fluid obtained by thoracentesis may be helpful for the diagnosis of pleural sarcoidosis when the diagnosis is already made by histological examination of more than one organ specimen.

## Introduction

Sarcoidosis is a systemic granulomatous disease of an unknown cause that may involve the lungs, lymph nodes, eyes, salivary glands, skin, liver, spleen, heart, nervous system, muscles, bone, and other organs [1]. A clinical diagnosis is made by the histological confirmation of non-caseous epithelioid granulomas in more than one organ specimen along with the exclusion of other known etiologies [1]. Sarcoidosis involves the lungs in more than 90% of cases [1]. Pulmonary sarcoidosis is characterized by the infiltration of activated T-cells bearing CD4 and macrophages [1]. When the CD4/CD8 lymphocyte ratio in bronchoalveolar lavage (BAL) fluid (BALF) is greater than 3.5, the sensitivity and specificity for the diagnosis of sarcoidosis are 53% and 94%, respectively, with a positive predictive value of 76% [1]. Serum soluble interleukin-2 receptor (sIL2R) and serum Krebs von den Lungen-6 (KL6) values are correlated with the number of total cells, lymphocytes and T-lymphocytes bearing CD4 in BALF, suggesting that these may reflect alveolitis induced by sarcoidosis [2]. Sarcoidosis can also involve the pleura: pleural sarcoidosis is observed in only 1.1% of out-patients with sarcoidosis [3] and in approximately 3% of all cases of sarcoidosis [4]. Pleural sarcoidosis is diagnosed clinically [3, 5, 6] or histologically by thoracoscopy [7, 8] or percutaneous pleural biopsy [3, 9].

Exclusion of tuberculosis and fungal disease is important for an accurate diagnosis [10]. Thoracoscopy may not always be required to determine the etiology of pleural effusion in patients with sarcoidosis when the diagnosis is already definitive by the histological analysis of other plural organ specimens. Here we report the case of a 64-year-old woman in whom analysis of the CD4/CD8 lymphocyte ratio in pleural effusion fluid obtained by thoracentesis was helpful for the clinical diagnosis of pleural sarcoidosis.

## Case presentation

**Fig. 1:**
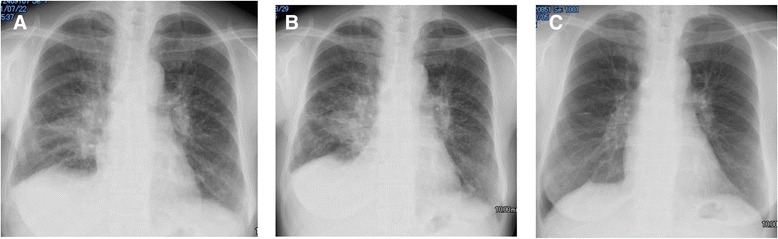
Chest radiography. **a** Before the first admission. **b** Before the initiation of corticosteroid therapy. **c** Five weeks after the initiation of corticosteroid therapy

A 64-year-old Japanese woman visited our hospital with an initial symptom of dyspnea on exertion. For the past 4 months she had shortness of breath when she walked approximately 100 meters or went up 10 to 20 steps of a staircase. She visited her neighboring hospital 1 month before visiting our hospital where she received a diagnosis of bilateral pleural effusions and seven sessions of thoracentesis; however, no clinical diagnosis could be made although her dyspnea on exertion was slightly improved by the frequent thoracentesis. When she visited our hospital for the first time, she felt dyspnea on exertion on the ground level after several hundred meters. Before she had dyspnea on exertion, she had not had any diseases including sarcoidosis nor had she received any continuous medications such as immunosuppressive drugs. She had no previous history relating pleural effusion, and no family history of sarcoidosis. She did not have any past history for opportunistic infections. A chest radiograph showed the enlargement of bilateral hilar shadows, reticulonodular shadows in bilateral lung fields, and dullness at the right costophrenic angle (Fig. 1a). Her serum angiotensin-converting enzyme (ACE) level was elevated to 44.2 IU/L. Her immunoglobulin G (IgG) and sIL2R levels were also elevated (Table 1). Bacterial culture for sputum resulted in negative findings for tuberculosis. Polymerase chain reaction (PCR) analysis also showed no tuberculosis in her sputum.

**Table 1:** Laboratory findings from an earlier visit to another hospital and first admission to our hospital

Hematology		Serology		Bronchoalveolar lavage analysis
WBC	4470/μl	CRP	0.79mg/dl	Cell numbers 3.5×10^5^/ml
Neu	60.3%	IgG	1893mg/dl	Cell fractionation
Lymphocyte	20.7%	ACE	44.2IU/L	Lymphocyte 33%
Monocyte	10.1%	sIL2R	3710U/ml	Neutrophil 1%
Eosinophil	4.0%	CEA	1.1ng/ml	Macrophage 66%
Basophil	1.2%	Blood gas analysis		CD4/8 13.43
RBC	464×10^4^/μl	pH	7.395	Bacterial culture negative
Hb	14.0g/dl	PaO_2_	72.9mmHg	Pleural effusion
Ht	41.8%	PaCO_2_	41.3mmHg	pH 7.5
Plt	22.4×10^4^/μl	HCO_3_	24.8mmol/L	Specific gravity 1.030
Biochemistry		Pulmonary function test		Rivalta 2+
TP	6.3g/dl	VC	1880ml	Protein 4.3mg/dl
Alb	3.3g/dl	%VC	82.3%	Alb 2.3mg/dl
AST	40IU/L	FEV1.0	1270ml	ADA 50.4IU/L
ALT	29IU/L	FEV1.0%	70%	CEA 0.8ng/ml
ALP	764U/L	PEF	3070mL/second	Cell numbers 882/μl
LDH	183IU/L	V50	1040mL/second	Cell fractionation
Crt	0.65mg/dl	V25	400mL/second	Lymphocyte 98%
FBS	90mg/dl	V50/V25	2.60	Neutrophil 2%
Na	141mEq/L	%DLCO	95.4%	CD4/8 ratio 5.62
K	4.5mEq/L	DLCO/VA	4580mL/minute/mmHg/L	PCR negative for tuberculosis
Cl	104mEq/L			Bacterial culture negative
Ca	9.2mg/dl			
IP	3.6mg/dl			

*ACE* angiotensin-converting enzyme, *ADA* adenosine deaminase, *Alb* albumin, *ALP* alkaline phosphatase, *ALT* alanine aminotransferase, *AST* aspartate aminotransferase, *Ca* calcium, *CEA* carcinoembryonic antigen, *Cl* chlorine, *CRP* C-reactive protein, *Crt* creatinie, *DLCO* carbon monoxide diffusion capacity, *FBS* fasting blood sugar, *FEV1.0* forced expiratory volume in 1 second, *Hb* hemoglobin, *HCO*_*3*_ bicarbonate, *Ht* hematocrit, IgG immunoglobulin G, *IP* inorganic phosphorus, *LDH* lactate dehydrogenase, *Na* sodium, *Neu* neutrophil*, PaCO*_*2*_ partial pressure of carbon dioxide in arterial blood, *PaO*_*2*_ partial pressure of oxygen in arterial blood, *PCR*, polymerase chain reaction, *PEF* peak expiratory flow, *Plt* platelets, *RBC* red blood cell*, sIL2R* soluble interleukin-2 receptor, *TP* total protein, *V50* and *25* expirtatory flow at 50% and 25% of vital capacity, respectively, *VA* alveolar volume, *VC* vital capacity, *WBC* white blood cell

**Fig. 2:**
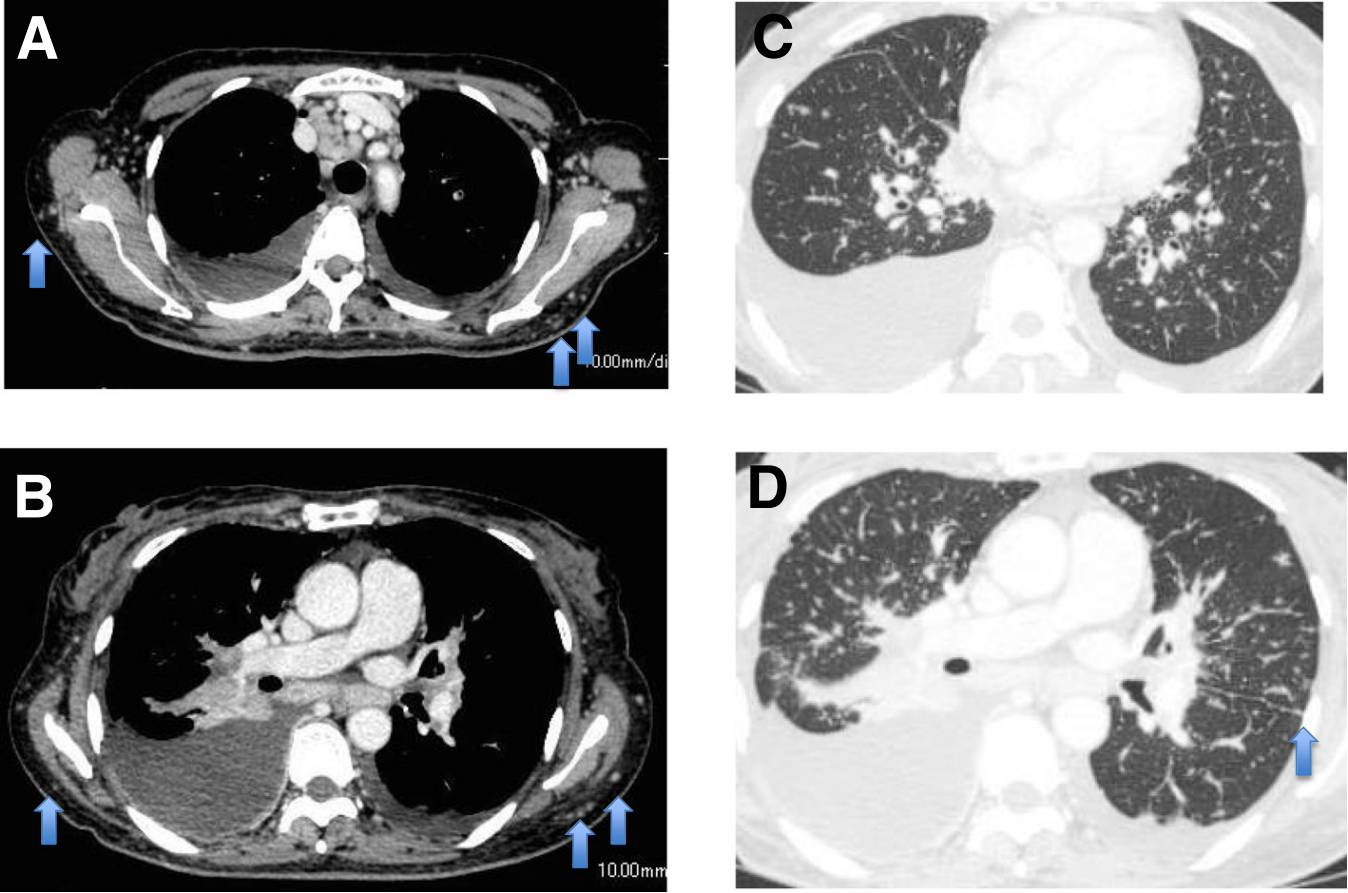
Chest computed tomography findings on admission. **a** and **b**: Multiple mediastinal and bilateral hilar lymphadenopathy, bilateral pleural effusion, and multiple small subcutaneous nodules indicated by *arrows*. **c** and **d**: Bilateral multiple small nodular shadows in the lungs and multiple small nodules along the interlobar pleura (*arrow*)

**Fig. 3:**
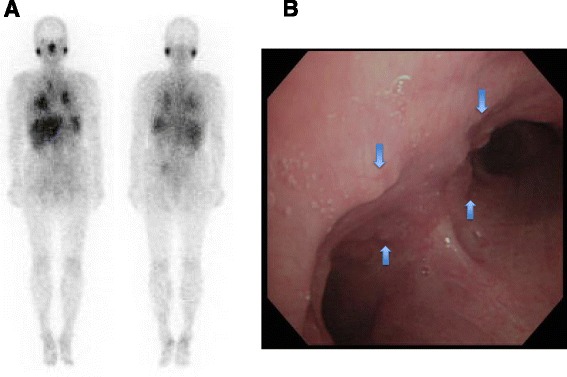
Gallium-67 scintigraphy and bronchoscopic examination. **a** Gallium-67 scintigraphy exhibits abnormal uptake in the right subclavicular area, mediastinum, bilateral hilum, bilateral parotid glands and spleen but no abnormal uptake in the heart. **b** A bronchoscopic examination reveals multiple small nodules on the surface of the bilateral bronchi (arrows). A carina is located at the center

**Fig. 4:**
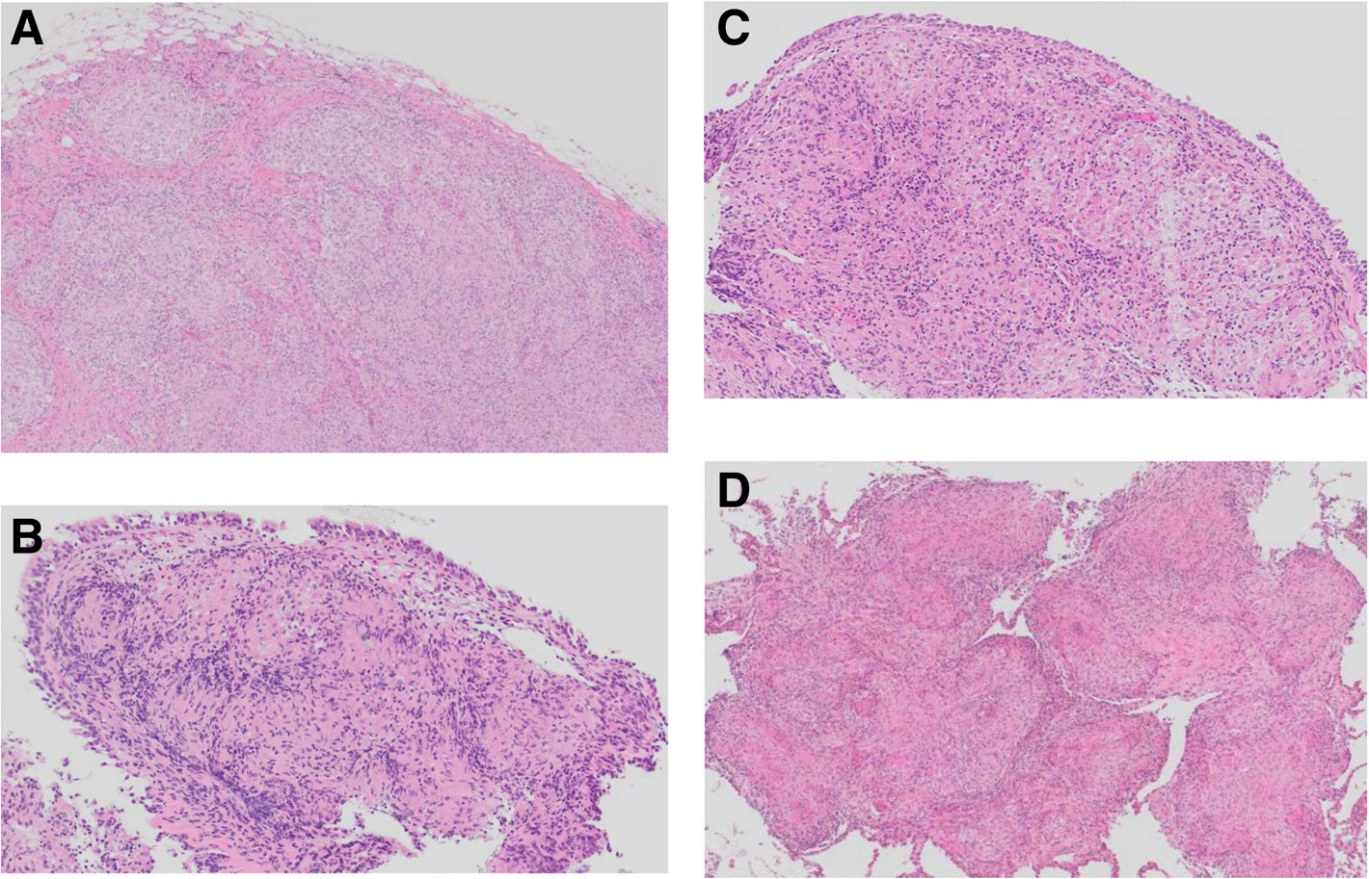
Histological analysis. All pictures are obtained at 20× magnification. **a** Hematoxylin and eosin staining of a subcutaneous nodule specimen. **b** Hematoxylin and eosin staining of a right mediastinal lymph node specimen. **c** Hematoxylin and eosin staining of an endobronchial nodule specimen. **d** Hematoxylin and eosin staining of a transbronchial lung biopsy specimen

Three weeks later, she was admitted to our hospital for diagnosis and treatment. Neither restrictive pulmonary function disorder nor remarkably reduced diffusing capacity of her lungs was observed. The 6-minute walk test revealed that she could walk up to 370 meters with a minimum blood oxygen saturation (spO_2_) of 93%, maximum pulse of 126 beats per minute and the worst modified Borg scale of perceived dyspnea of 1. Chest computed tomography (CT) showed multiple skin nodules, bilateral hilar and mediastinal lymphadenopathy, bilateral pleural effusion, and multiple small nodules in both lungs and along the interlobar pleura (Fig. 2a-d). Pulmonary embolism was not observed in chest CT. Multiple subcutaneous nodules were palpable in her neck, back, and bilateral arms. Gallium-67 scintigraphy exhibited abnormal uptake in the right subclavicular area, mediastinum, bilateral hilum, bilateral parotid glands, and spleen, but no abnormal uptake in her heart, suggesting sarcoidosis or malignant lymphoma (Fig. 3a). A tuberculin test was negative. A skin biopsy specimen was obtained and bronchoscopy was performed twice, including endobronchial ultrasound-guided transbronchial needle aspiration (EBUS-TBNA), BAL, and transbronchial lung biopsy. Thoracentesis was also performed. Histological analysis of a resected subcutaneous nodule from her left upper arm exhibited non-caseous epithelioid granuloma (Fig. 4a). Ziehl–Neelsen staining and Grocott staining tested negative. Histological analysis of a right mediastinal lymph node (station 4R) obtained by EBUS-TBNA also showed a non-caseous epithelioid granuloma; malignant lymphoma was ruled out (Fig. 4b). The aspiration sample of the lymph node revealed negative findings for bacteria including tuberculosis. In addition, PCR analysis for detection of tuberculosis, *Mycobacterium avium* and *Mycobacterium intracellulare* were negative. Bronchofiberscopy revealed multiple small nodules in both the main bronchi (Fig. 3b), and histological analysis of an endobronchial nodule and a transbronchial lung biopsy specimen from the right upper lobe revealed non-caseous epithelioid granulomas (Fig. 4c and d, respectively). The transbronchial lung biopsy specimen also showed negative Ziehl–Neelsen and Grocott staining. BALF obtained from the right middle lobe exhibited an increased number of lymphocytes and an increased CD4/CD8 lymphocyte ratio of 13.43 (Table 1). Bacterial culture, including that of *Mycobacteria*, was negative. On the basis of these findings, a final diagnosis of sarcoidosis was made.

Thoracentesis on the right side revealed exudative pleural effusion fluid. Cell fractionation of the fluid showed the predominance of lymphocytes without any malignant cells and an increase in the CD4/CD8 lymphocyte ratio to 5.62 (Table 1). Her adenosine deaminase (ADA) level in her right pleural effusion was 50.4IU/L. However, bacterial culture including that for *Mycobacteria* was negative and PCR analyses for detection of tuberculosis, *Mycobacterium avium* and *Mycobacterium intracellulare* were negative. Examinations of fundus, electrocardiography and echocardiogram revealed no abnormality. Collectively her lungs, skin, pleura, and, possibly, the parotid glands and spleen were involved in sarcoidosis.

**Fig. 5:**
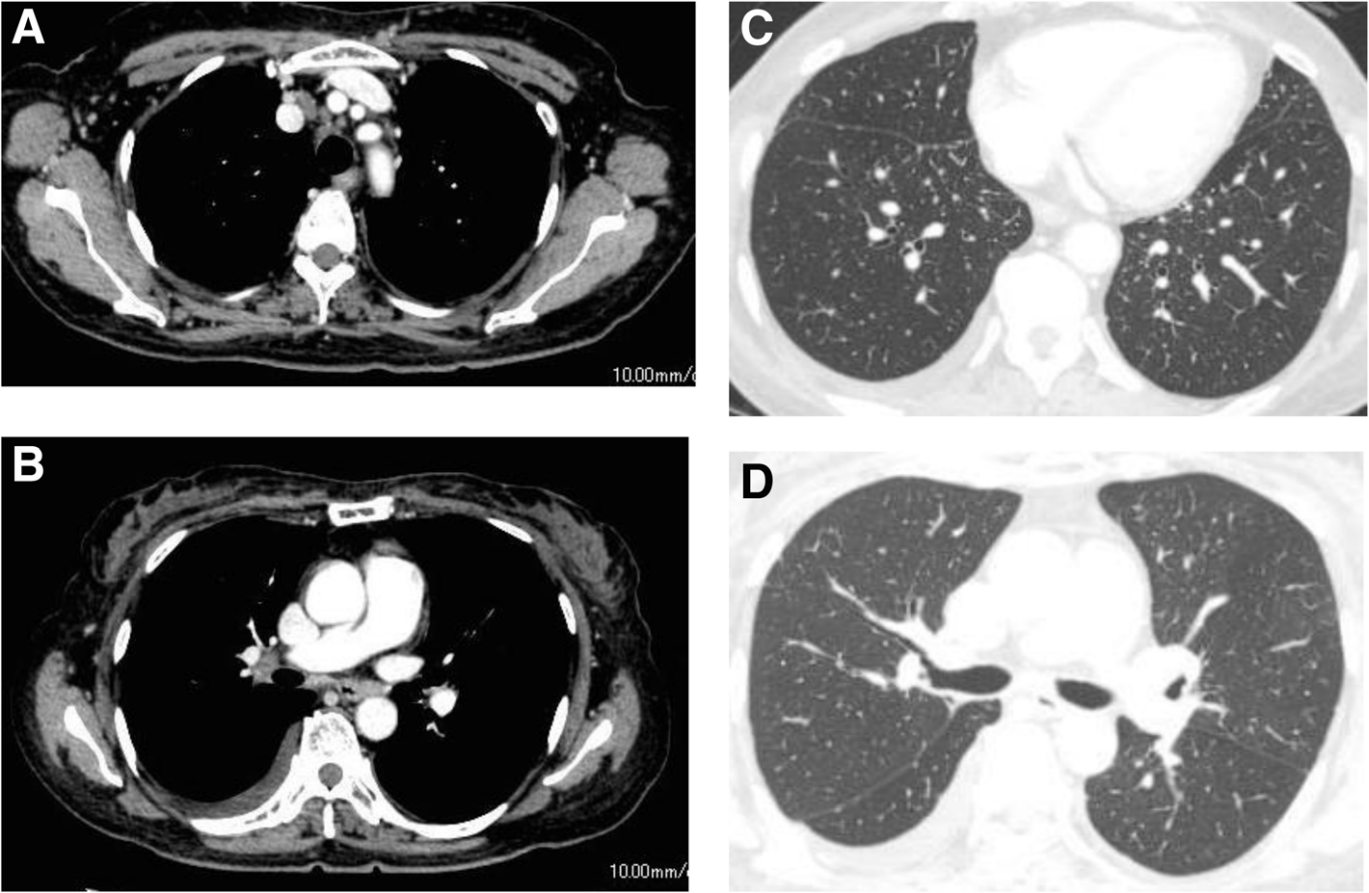
Chest computed tomography findings after 5 weeks of corticosteroid therapy. **a**-**d** The multiple mediastinal and bilateral hilar lymphadenopathy, bilateral pleural effusion, small nodules in the lung and the interlobar pleura, and small subcutaneous nodules have regressed

She was discharged 5 weeks after the first visit and was followed up for the spontaneous resolution of the bilateral pleural effusion. However, no improvement was observed and pleural effusion increased (Fig. 1b). The 6-minute walk test revealed that she could walk up to 315 meters with a minimum spO_2_ of 90%, maximum pulse of 116 beats per minute and the worst modified Borg scale of perceived dyspnea of 4. In addition, her initial symptom, which was induced by walking approximately 100 meters or going up 10 to 20 steps of a staircase, seemed to be severe and was partially improved by frequent thoracentesis. Based on these findings, corticosteroid therapy with a daily dose of 30mg prednisolone was initiated 8 weeks after the first visit. Thirteen weeks later (5 weeks from the initiation of corticosteroid therapy), under a daily dosage of 25mg of prednisolone, a chest radiograph showed decreased pleural effusion (Fig. 1c), and CT revealed regression of multiple skin nodules, mediastinal and bilateral hilar lymphadenopathy, multiple small nodules in her lungs and the interlobar pleura, bilateral pleural effusion, and splenomegaly (Fig. 5a-d). The clinical course of pleural effusions corroborates the diagnosis of pleural sarcoidosis. Three years later, she received 2.5mg of prednisolone per week without any severe adverse events; a chest CT showed only reduced small nodules in her lungs, lymphadenopathy, and subcutaneous nodules and no pleural effusion.

## Discussion

Pleural sarcoidosis is believed to be rare. Huggins *et al*. reported that 2.8% of out-patients showed pleural effusion, although a histological diagnosis was made only in 1.1% [3]. In several reported cases, pleural sarcoidosis is histologically diagnosed by thoracoscopic surgery [7, 8]. Although percutaneous biopsy is useful in some cases [3, 9], it is not always helpful for diagnosing pleural sarcoidosis [11]. In our case, biopsy specimens obtained from three organs, namely the skin, lymph nodes and lungs, all exhibited non-caseous epithelioid granulomas, thus supporting a diagnosis of sarcoidosis. However, the ADA level in the right pleural effusion was elevated in our case. It seemed to be difficult to conclude that the high level of ADA was due to pleural sarcoidosis before treatment. The ADA level in pleural effusion is well known to be elevated by tuberculosis [12]. When the ADA level in pleural effusion is cut off at 40IU/L, then all of pleural tuberculosis exceeded 40IU/L, while 2.5% of non-tuberculosis disease exceeded 40IU/L. ADA levels of two cases of sarcoidosis included in the non-tuberculosis group were less than 40IU/L [12]. We thought that tuberculosis should be carefully ruled out. The histological analyses by Ziehl–Neelsen staining, the *Mycobacterium* cultures, and PCR analyses did not support tuberculosis. Fungal infection was also ruled out by histological examination and bacterial culture of both BALF and pleural effusion fluid. We thought that thoracoscopy was too invasive for the patient as we regarded this case met the diagnosis criteria of sarcoidosis. We also excluded malignant lymphoma by histological examination of a EBUS-TBNA specimen because her serum sIL2R level was elevated at the first visit. sIL2R is not only a marker of malignant lymphoma but is also elevated when a T-cell is activated [13]. Miyoshi *et al*. reported the elevation of sIL2R in sarcoidosis with active alveolitis [2]. We finally diagnosed pleural sarcoidosis on the basis of the identification of small nodules along the interlobar pleura, lymphocytic predominance, and an increased CD4/CD8 lymphocyte ratio in pleural effusion fluid. The CD4/CD8 lymphocyte ratio in pleural effusion induced by tuberculosis is reported to be 3.1±1.1 (mean ± standard deviation) [14]. The CD4/CD8 lymphocyte ratio of the right pleural effusion in our case showed 5.62, which was higher than the ratio of mean +2 standard deviation value of pleural tuberculosis reported by Aguiar *et al*. [14]. Corticosteroid therapy resulted in regression of both small nodules along the interlobar pleura and bilateral pleural effusion, consistent with the findings in pleural sarcoidosis. We have carefully followed up for more than 3 years after the initiation of corticosteroid therapy. Bilateral pleural effusions disappeared and have not increased. We regard that tuberculosis and fungal infection were completely excluded. Therefore we concluded that this was a case of pleural sarcoidosis with the elevation of CD4/CD8 lymphocyte ratio in pleural effusion. The increased CD4/CD8 lymphocyte ratios in BALF and pleural effusion fluid suggested that T-lymphocytes bearing CD4 are activated not only in the lungs but also in the pleura, which may be helpful for diagnosis of pleural sarcoidosis. When patients with sarcoidosis and pleural effusions are already diagnosed histologically in plural organ specimens, the increased CD4/CD8 lymphocyte ratio in pleural effusion fluid obtained by thoracentesis may be a minimally invasive substitute for thoracoscopic surgery for the diagnosis of pleural sarcoidosis.

Pleural involvement of sarcoidosis is considered to be an indication for corticosteroid therapy in recurrent or symptomatic cases [10, 11]. Asymptomatic pleural effusion may resolve spontaneously [10]. The effects of corticosteroids for pleural sarcoidosis were satisfactory in our case. This clinical course supports the findings in a previous report that pleural sarcoidosis is susceptible to corticosteroid therapy [11].

## Conclusions

In conclusion, we report a case in which pleural sarcoidosis was diagnosed on the basis of lymphocytic predominance and an increased CD4/CD8 lymphocyte ratio in pleural effusion fluid obtained by thoracentesis. This technique may be useful for the diagnosis of pleural sarcoidosis when sarcoidosis is already diagnosed histologically in biopsy specimens from more than one organ.

## Consent

Written informed consent was obtained from the patient for publication of this case report and any accompanying images. A copy of the written consent is available for review by the Editor-in-Chief of this journal.

## Abbreviations

ADA: Adenosine deaminase
BAL: Bronchoalveolar lavage
BALF: Bronchoalveolar lavage fluid
CT: Computed tomography
EBUS-TBNA: Endobronchial ultrasound-guided transbronchial needle aspiration
PCR: Polymerase chain reaction
sIL2R: Soluble interleukin-2 receptor
spO_2_: Blood oxygen saturation
